# Meta-Analysis of Individual and Environmental Factors that Influence People’s Addiction Tendencies

**DOI:** 10.5812/ijhrba.5330

**Published:** 2012-11-26

**Authors:** Saideh Safari Hajat Aghaii, Ayoub Kamaly, Mehdi Esfahani

**Affiliations:** 1 Department of clinical Psychology, Isfahan University, Isfahan, IR Iran

**Keywords:** Meta-Analysis, Behavior, Addictive, environmental

## Abstract

**Background:**

In recent years, many studies have been conducted to establish the causes of people’s tendency to become addicted and researchers have also tried to determine the amount, importance and role of each individual and environmental factor.

**Objectives:**

With regard to the inconsistencies in previous research results, this study aims to use meta-analysis in order to integrate the results of different studies and investigate the impact of environmental and personal factors in people’s proclivity to addiction.

**Materials and Methods:**

This meta-analysis uses the Hunter and Schmidt approach. For this purpose, 16 out of 32 studies which were acceptable in terms of their methodology, and had been conducted during an eight year period (2003 - 2010), were selected. A meta-analysis was conducted on the articles which had been collected using a standard checklist via; the internet, in person, telephone and e-mail, from universities and research centers across the country. After summarizing the results of the studies, effect sizes were calculated manually and combined based on a meta-analysis, and interpreted in accordance with a Cohen’s table.

**Results:**

After data collection, results showed that the effect size of environmental factors in people’s tendency to addiction was 0.61 (P ≤ 0.00001), and the effect size of individual factors in people’s tendency to addiction was 0.45 (P ≤ 0.03).

**Conclusions:**

According to Cohen’s table size, the effects were evaluated as average to high for the environmental factors and low to moderate for the individual factors in the tendency to become addicted.

## 1. Background

The problem of drug abuse is one of four global crises and a major social problem in many countries, which is closely linked with other aspects of the economy and culture (1). Addiction is a biological, psychological and social disease that affects more than 15% of the population over 18-years-of-agein America. Approximately two thirds of the population is addicted to alcohol and one third are drug users (2). According to official statistics, in 2004, the number of drug users in Iran was 4 million, with more than 2.5 million regular consumers (3). In the Diagnostic and Statistical Manual of Mental Disorders (DSM IV), the American Psychiatric Association defined drug addiction or drug abuse as a maladaptive pattern of substance use that leads to significant distress (4). In addition the World Health Organization (WHO) defined substance abuse as, ‘use of harmful and dangerous drugs like alcohol and narcotic drugs’ (5). Three characteristics of addiction are physical or physiological dependence, psychological dependence and drug tolerance. Physical or physiological dependence appears following cessation of the drug. Psychological dependence which is called ‘the habit,’ is an intense and continuous or intermittent desire for the drug to avoid discomfort or the effects of a hangover (6). The serious consequences of addiction run broad and deep; health, family life, economy, security and the cultural development of society are all affected. Inhibition of development, political stability and threats to the process of democracy in communities, are symptoms which are attributed to addiction problems (7). Drug abuse and drug dependence are like fatal diseases and they can have similar out comes. Often the result of this dependence shows itself in physical damage, behavioral problems, and relationships with other people. Substance abuse is used in a range of circumstances and in addition to recreational purposes, it is also used to cover life failures. In the past few years, many changes have been made in the field of substance abuse. Adolescent addiction in the metropolises has become a major problem and this has caused considerable damage to their communities (8). According to a United Nations report, 220 million people worldwide are living with substance abuse. Many people require drugs daily like opium, heroin, grass, hashish, marijuana, cocaine, and morphine. Approximately 160 million people use hashish, 14 million cocaine, 9 to 12 million opium and 50 million use different kinds of chemical drugs (9). Geographically, Iran borders Afghanistan, the largest producer country of opium and natural opiates that produces 3000 tons of drugs annually, making Iran the biggest consumer of opium in the world (10). The estimates show that the amount of direct and indirect economic and social damage from drug trafficking in the country is $700 billion annually (11). Addiction is a physical, psychological, social, and spiritual illness (12), and there are numerous pre-addiction elements which play fundamental roles in its formation. Individual factors include spiritual poverty, depression, illness, pleasure-seeking, lack of confidence, independence, lack of character development, hopelessness, escape from life’s problems, and low education. Environmental factors including drug addiction of one or more family members, family conflict, lack of child supervision by parents, parental unemployment (especially fathers), and parents with low literacy. Social factors include; school, friends, unhealthy entertainment, unemployment, lack of social acceptance, cultural poverty, population growth, and uncontrolled migration. Geographic and economic factors include; residence near drug traffic routes, ease of access to drugs, poverty, economic crisis, and unemployment (13). Studies that have been conducted within Iran on factors which have an effect on addiction include research by Jena Abadi et al. (14), Keldi et al. (15), Forood in et al. (16), Ebrahimbi Salami et al. (17), Ghaemi et al. (18), Akbarizardkhaneh et al. (19), Zeinali et al. (20), Zeinali et al. (21), Ghaziejad et al. (22), Dortaj (23), Alavi (24), Mirzaii et al. (25), Abdolrasooli (26), Nastizaii et al. (27), Aminian et al. (28), and Zargar et al. (29).

## 2. Objectives 

With regard to inconsistencies in previous research results, this study aims to use meta-analysis by integrating the results of different studies to investigate the impact of environmental and personal factors, in people’s tendency to become addicted.

## 3. Materials and Methods

In this study meta-analysis methods have been used in order to conduct the research. The concept of meta-analysis was first proposed in 1976 by Glass (30). The basic principles in a meta-analysis are measuring the effect size of the various researches, and translating those to a common matrix (general matrix), then combining them in order to acquire the mean of the effect (31). The statistical population of this study was all researches, thesis, and published scientific research articles, concerning the issue of individual and environmental factors that influence people’s tendency to become addicted, which had been conducted during an eight year period (2003 - 2010). All of the 32 studies mentioned in this research had good sample sizes and were favorable in terms of the reliability and stability of their measuring instruments and sampling methods. Researches and studies that were acceptable methodologically and met the criteria of internal consistency were used, and this consisted of 16 cases. The criteria of internal consistency included: 1) Having acceptable methodology (hypothesizing, research methodology, statistical population, sample size, sampling, statistical hypotheses, method of statistical analysis, and accuracy of statistical calculations). 2) The issue of research or study was individual and environmental factors influencing a tendency to addiction, 3) Research or study conducted in a group research frame work (neither case study nor single test). A checklist of content analysis was used in order to choose thesis and research papers that had the criteria of internal consistency and relevance, in order to extract the needed information for meta-analysis. The present meta-analysis was conducted in seven steps: 1) defining the variables, 2) exploring database and informational sources, 3) collecting the research reports, 4) measuring the effect size from each research, 5) combining the amount of effect sizes from all the examined researches, 6) determining significant levels from the combined researches, 7) and finally determining other influence variables (mediator variables) on the tendency to addiction. All of the calculations were performed manually and no software was used in this study. In order to calculate the mean of the effect size (r¯), the approach of Hunter and Schmidt was used. The steps of this method are shown in the following section:

**Figure:**
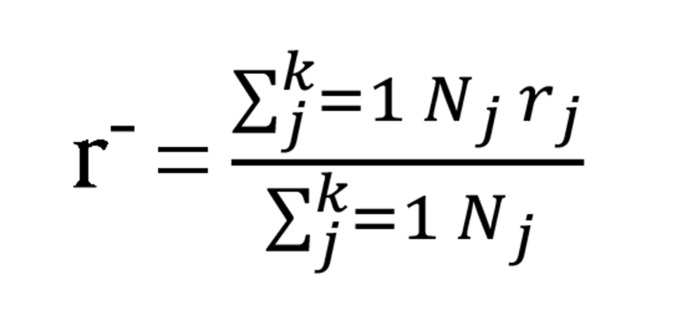


As indicated in this formula, in order to calculate the amount of the total effect size (r¯), we needed to determine the amount of ther’ effect. We can calculate the size of the ‘r’ effect through formulas based on x^2^, T and F (30).

These formulas include the following forms:

**Figure:**
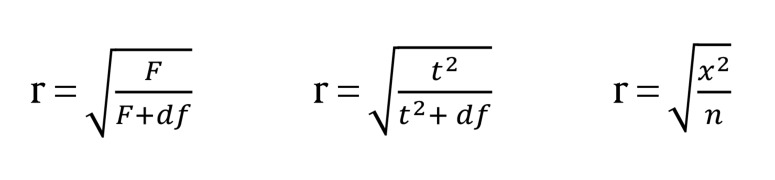


However, sometimes this statistical information was not mentioned in the preliminary resource. If you possess the sample size and significance level, then you can apply the following formula to estimate the effect size, regardless of the involvement of meaningful tests (30).

**Figure:**
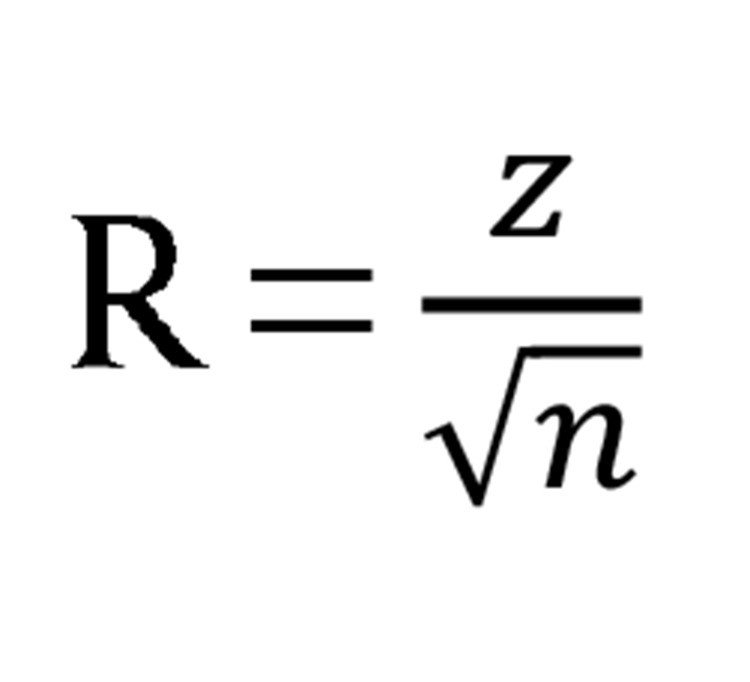


Finally in order to combine significant levels of studies and acquire overall significance levels, the approach of Hunter and Schmidt has been used and this formula has been indicated in the following section:

**Figure:**
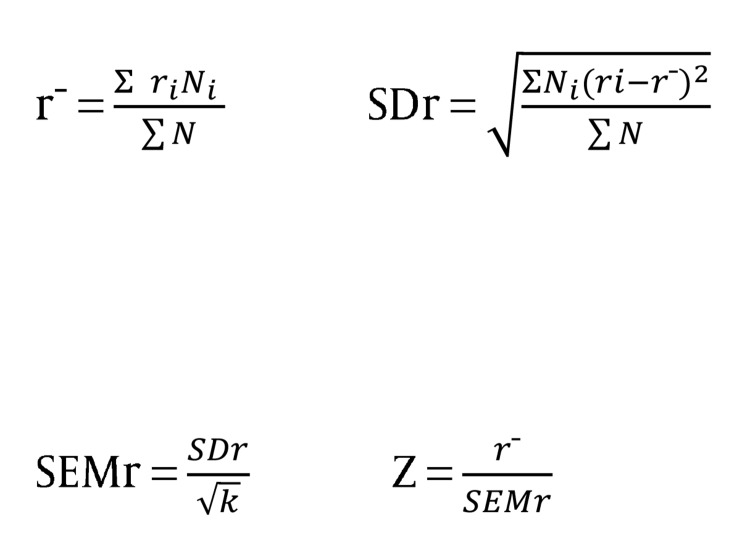


In addition, the effect amount of the mediator variable was calculated from the proportion of sampling error variance to total variance. That method of calculating mediator variable ‘SEV, V_total_’ is indicated in the following section:

**Figure:**
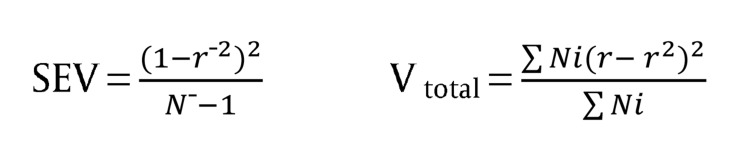


## 4. Results

In order to achieve the purpose of this study, this section includes three sets of findings. The first set includes descriptive information of the collected researches through searches from the internet, journals, libraries, and colleges which are indicated in (Table 1).

**Table 1.** Descriptive Information Used in the Meta-Analysis Research

	Source	Sample Size	Instrument
**Jena Abadi (14)**	Journal of Educational Psychology Studies	100	Emotional self-awareness scale, Impulse control scale, Bar-on' emotional intelligence (A.P.S)
**Keldi (15)**	Social Welfare Quarterly	227	Rotter's scale internal and external control
**Frooedin (16)**	Social Welfare Quarterly	198	Interview, Researcher's questionnaire
**Ebrahim Bay Salami (17)**	Social Welfare Quarterly	100	Review documents
**Ghaemi (18)**	Knowledge and Research in Applied Psychology, Khorasgan Islamic Azad University	200	Researcher's questionnaire to assess individual attitudes to drug addiction
**Akbari Zardhaneh (19)**	Iranian Psychologists Quarterly	296	Defense style questionnaire, Addiction Acknowledgment Scale
**Zeinali (20)**	Iranian Journal of Psychiatry and Clinical Psychology	240	Addiction potential scale
**Zeinali (21)**	Family Research Quarterly	304	Addiction Potential Questionnaire students version (parenting styles question (PSQ))
**Ghazi Nejad (22)**	Iranian Journal of Social Issues	384	Addiction potential scale
**Dortaj (23)**	Social Sciences Quarterly	380	Causes of drug abuse and drug dependence questionnaire
**Alavi (24)**	Journal of Chinese Clinical Medicine	283	Effect of self-esteem on substance-abuse, theft and prostitution
**Mirzaii (25)**	Iran Journal of Nursing	200	Structured interview
**Abdolrasooli (26)**	Cultural Research Quarterly	374	-
**Nastizaii (27)**	Journal of Urmia Nursing And Midwifery Faculty	200	Interview, researcher's questionnaire
**Aminian (28)**	Journal of Family research	426	Researcher's questionnaire
**Zargar (29)**	Journal of Education and Psychology, Ahvaz University	489	Readiness questionnaire to addiction, Marital satisfaction and religiosity questionnaire, Arendt's sensation seeking questionnaire, Psychological hardiness questionnaire, Assertiveness questionnaire

In the researches reviewed, we extracted and classified the influencing variables (independent variables) on addictive behaviors and the tendency to abuse drugs (Table 2). These reviewed researches included 41 individual variables and 35 environmental variables. Each of the independent variables from the selected research hypotheses are offered based on the frequency and presence of each of the hypotheses. The third set is the findings that were collected from research samples during the data analysis step and these are indicated in Table 3. In Table 4 the results of the effect sizes of independent variables about the hypotheses of reviewed researches are shown, along with the results of tests which determined mediator variables.

**Table 2.** Individual Factors Used in the Meta-Analysis

	Frequency	Statistics	Cohen's d	Pearson Correlation Coefficient = r
**Emotional self-awareness**	2	r	0.85	-0.391
**Impulse control**	1	r	1.22	-52.0
**Age**	8	x^2^ = 14.59	0.270	0.56
**Education**	8	x^2^ = 9.48	0.41	0.20
**Marital**	8	x^2^ = 19.08	0.28	0.34
**Occupation**	10	x^2^= 14.78	0.25	0.52
**Frequency of withdrawal**	1	x^2^= 8.91	0.19	0.39
**Religious attitude**	7	r	0.54	-0.26
**Social commitment**	2	*t* = 4.879	0.98	0.44
**Social control**	5	*t* = -3.225	0.29	0.98
**Knowledge of drugs and narcotics**	6	r	0.43	-0.21
**Consider addiction as a treatable, benign problem**	3	r	0.19	0.39
**Defensive mechanisms developed**	1	*t* = 3.7	0.45	-0.22
**Emotional intelligence**	3	r	-0.28	0.58
**Applying emotional employment**	2	r	0.16	-0.08
**Emotional regulation**	1	r	-0.15	0.30
**Neurotic defense mechanisms**	1	*t* = 2.34	0.39	0.19
**Immature defense mechanisms**	1	*t* = 0.37	0.24	0.12
**Personality characteristics**	4	r	9.85	0.98
**Thoughts and beliefs**	1	r	6.86	0.96
**Behaviors**	3	r	3.71	0.88
**Lifestyle**	2	r	6.08	0.95
**Feelings and emotions**	2	r	4.69	0.92
**Material deprivation**	8	r	0.80	0.37
**Economic factors**	9	x^2^ =196.99	2.08	0.72
**Duration of leisure**	1	x^2^ = 27.44	0.65	0.31
**Duration of participate in parties**	3	x2= 0.169	0.04	0.02
**Physical condition**	1	x^2^ = 107.5	2.14	0.73
**Mental status**	2	x^2^= 84.5	1.71	0.65
**Negative feelings**	1	x^2^= 52.11	0.80	0.37
**Religious**	4	x^2^= 17.66	0.43	0.21
**Self-esteem**	5	x^2^ = 36.89	0.65	0.31
**Associated factors**	1	x^2^ = 9.21	0.43	0.21
**Internal monitoring**	2	*t* = -5.643	0.54	0.26
**Seeking emotion**	2	r	0.77	0.36
**Need to belong**	3	r	0.32	0.24
**Assertiveness**	3	r	0.18	-0.09
**Psychological hardiness**	2	r	0.72	-0.34
**Marital satisfaction**	3	r	1.32	-0.55

**Table 3.** Environmental Factors Used in the Meta-Analysis

	Effect Size			
	Frequency	Statistics	d	R
**Influence of friends**	13		0.63	0.30
**Addiction of family members**	12		0.24	0.12
**Father's addiction**	12	*P* = 0.007	0.43	0.21
**Norm – breaker friends**	8	*P* = 0.003	0.43	0.21
**Addiction in relatives**	10	*P* = 0.003	0.43	0.21
**Effect of information sources on drug awareness**	7	r	0.43	-0.21
**Social and family relationships**	14	r	6.86	0.96
**Authoritative parenting style**	1	r	0.7	-0.33
**Authoritarian parenting style**	1	r	0.39	0.19
**Free transition parenting style**	1	r	0/04	0/02
**Parenting style disregard**	1	r	0.22	0.11
**Rejected parameters and conditions**	3	r	0.82	0.38
**Subculture approves of addiction**	3	r	1.39	0.57
**Rejected predictive factors**	5	r	0.70	0.33
**Rejected transition period factors**	1	r	0.850	0.39
**Rejected transition period factors**	2	r	0.18	0.09
**Lack of social participation**	2	r	0.72	0/34
**Lack of normative integration**	2	r	1.15	0.50
**Addiction as a leisure time activity**	3	r	0.49	0.24
**Relationship with peers who approve of addiction**	14	r	0.52	0.25
**Cultural–educational factors**	2	x^2^ = 98.83	1.19	0.51
**Family factors**	13	x^2^ = 255.1	2.87	0.82
**Legal factors**	2	x^2^ = 150.82	1.62	0.63
**Social factors**	4	x^2^ = 25.63	0.63	0.30
**Treatment conditions**	1	x^2^ = 30.42	0.85	0.39
**Social pressure from others**	14	x^2^ = 20.64	0.47	0.23
**Addicted friends**	14	x^2^ = 9.21	0.43	0.21
**Contaminated environment**	5	x^2^ = 6.63	0.37	0.18
**Rate of response to social needs**	4	*t* = -3.772	0.54	0.26
**Cultural factors**	3	*t* = -3.087	6	0.14
**Lack of social rights**	2	r	0.72	0.34
**Friends status**	12	x^2^ = 7.05	0.03	0.15
**Relatives status**	12	x^2^ = 7.05	0.30	0.15
**Social status**	2	x^2^ = 82.3	1.05	0.60
**Family status**	12	x^2^ = 128.7	2.67	0.80

**Table 4.** Effect Size of Individual and Environmental Factors and the Effect of Modulatory

	Mean of Effect Size (d)	Mean of Effect Size (d)	Significance Level	SEV/V_total_
**Individual factors**	0.45	0.22	0.03	0.38
**Environmental factors**	0.61	0.29	0.00001	0.53

## 5. Discussion

The basic purpose of a meta-analysis in internal studies is in terms of the factors that influence an addiction tendency. These could lead to an overall view about the study results on the desired subject, by combining and integrating results from previous studies. Moreover, this could lead to the identification of different, similar and opposite findings and results from previous researches and determine the amount of the effect and the influencing factors on addiction and the tendency to abuse drugs. The results of Table 3 indicate that the mean of the acquired effect size of the environmental factors, in terms of a tendency to addiction, was 61% (P ≤ 0.00001), and the level of the individual factor’s effect size in terms of tendency to addiction was 45% (P ≤ 0.03). The effect size indicated the amount or degree of the phenomena present in society. Based on Cohen’s table, the changing Cohen effect size of the environmental factors evaluated with the individual factors was low to moderate. This means that the results of the meta-analysis indicate that there is a greater relationship between environmental factors than individual factors, in the tendency to become addicted in Iran. It was concluded that the difference between individual and environmental factors on the tendency to become addicted is significant (H0). The other important section of meta-analysis is discovering mediator variables which interfere in the relationship between two studied variables. Researchers realize these mediator variables through mathematical equations, and in regard to the results of later studies, mediator variables can then be predicted. Indeed, if sampling error is 75% of the total variation or more, the variation is related to evaluating error, and the measured relationship is not influenced by the mediator variable. In the present study, Sev/v _total_ was calculated as 0.75 ≥ 0.53 for the environmental variables and 0.75 ≥ 0.38 for the individual variables. This means that the effect of the mediator and influencing variables on the environmental and individual variables is overt. Therefore, with regard to later studies and measurements of mediator variables, it is concluded that these two factors play an integrative role on the tendency to addiction. Results of this study also indicated that environmental factors influenced the tendency to addiction more than individual factors. The following suggestions are offered based on these results:

1) Preparing comprehensive prevention approaches by combining the best components of currently available prevention methods. In this regard, it is suggested that users of prevention models and experts in welfare issues, base their approach on the results of such meta-analysis about prevention in Iran and in other countries. They should also be based on a strategic Naida design which is a 5-year strategic plan to prevent substance abuse that is research-based approach and with the help of combined strategies focuses on reducing substance abuse and high risk behaviors In all groups, especially youth, creating a suitable model for the individual’s needs, along with the cultural, social and political circumstances of the target community in an effort to raise awareness and the prevention of substance abuse.

2) Efforts to reinforce family relationships, parental relationships, parent - child relationships, and parent-relatives’ relationships, in order to improve poor relationships and create healthy relationships.

3) Informing parents about potential problems and issues concerning the needs of the growing period, especially in adolescence and younger children is important. They should be informed that providing intimate, close relationships between parents and their children, as well as listening to their issues and problems, can lead to the prevention of inappropriate relationships. Based on these strategies, parents are able to prevent their children from developing inappropriate relations.

4) Parents adopting methods for increasing their children’s confidence and teaching them life skills will help their children to resist against the pressures of norm-breaking groups.

5) Considering the important role of friends and peers in the formation of values, attitudes, and behaviors, it is important to avoid the formation of relationships within appropriate friends.

6) Increasing social controls (either formal or informal controls) in order to ensure compliance with rules.

7) Offering necessary information about the role played by some advertisements which promote methods of leaving addiction, as they may lead to a tendency to become addicted.

8) Conducting special workshops and seminars that are attractive to parents and their children, in order to warn them about the dangers of drugs and changing their attitudes toward drugs.

9) Filling the gap between practice and study results. This issue needs further studies and research about how to promote, accept, and internalize effective prevention perspectives in schools, universities, and societies.

Therefore, the design and conduct of comprehensive and continuous prevention programs should be based on the principles of mental health, and there is also a need for the increased involvement of cultural bodies such as; the Ministry of Education, Ministry of Culture and Islamic Guidance, and the IRIB medical services). However, it is important to keep in mind that community health also needs social and economic support from groups and organizations in the community, in addition, a theory cannot be helpful solely for addiction, as it should be used as a holistic approach for personal and social development as well. Accordingly, access to drugs, social acceptance and peer pressure, are the main factors that affect drug use in the first instance. Nevertheless, there are probably other factors such as the individual’s personality, and biological characteristics, which also play an important role in understanding the drug’s effects and rate of change in the nervous system due to frequent use. Finally, for a definitive answer to the question, ‘what causes people’s tendency to become addicted?’ It is essential that further meta-analyses are carried out with more research on the role of the factors which have not been studied here. Although in this meta-analysis all relevant studies (research, thesis and papers) were evaluated in terms of their content and we did not rely only on published studies or studies, which were readily available through an internet search. The limitations of such a meta-analysis lie ingraining access to the resources and research which have been conducted in such a specific area, and this includes that they have been published. Considering that the present study is the first academic meta-analysis aimed at the problem of drug abuse, it is recommended that future researchers conduct more research in this area with larger sample sizes and with regard to different age groups, gender differences and pay particular attention to individuals who have experienced stopping drug use, one or more times. It is also recommended that separate and comparison meta-analyses of biological, psychological and social factors are carried out. Each component of them an infectors (environmental and personal) should be examined in a more specific and separate meta-analysis. Moreover future research should not ignore the role of protective factors which mitigate against people’s tendency to abuse drugs.
